# Value in app store metadata and user reviews: A dual perspective on quality of Parkinson’s and dementia apps

**DOI:** 10.1371/journal.pdig.0001730

**Published:** 2026-10-01

**Authors:** Isabel Schwaninger, Paria Ighanian, Liyousew Borga, Marijus Giraitis, Petra Hoogendoorn, Jochen Klucken

**Affiliations:** 1 Digital Medicine, Luxembourg Centre for Systems Biomedicine (LCSB), University of Luxembourg, Esch-sur-Alzette, Luxembourg; 2 Centre Hospitalier de Luxembourg (CHL), Strassen, Luxembourg; 3 National eHealth Living Lab, Public Health and Primary Care Department, Leiden University Medical Center, Leiden, Netherlands; University of Pittsburgh School of Medicine, UNITED STATES OF AMERICA

## Abstract

App stores provide a range of health applications for people living with Alzheimer’s disease (AD) and Parkinson’s disease (PD). Users like patients can access information from both manufacturer-provided app descriptions and metadata, as well as from user reviews. However, navigating this information to identify value-generating apps of high quality remains challenging. From a health technology assessment (HTA) standpoint, quality is defined by evidence-based medicine, safety, and user-centeredness. To explore the potential value of app store information available to patients, we conducted a descriptive, mixed-methods analysis of unstructured app metadata and user feedback from the Apple App Store and Google Play Store (as of May 2024). We analyzed 1,237 AD/PD-related apps which included 50 duplicates (resulting in 1,187 apps), utilizing descriptive statistics, content analysis, large language model-supported exploratory classification, and a topic modeling approach. In total, only about 2% of the apps claimed to be certified medical devices. Moreover, 24% of Apple apps and 14% of Google apps were in the “Medical” app store genre, among which we found that 63% of Apple and 54% of Google apps were patient-facing. We manually categorized patient-facing apps predominantly under “Care Support,” followed by “Health & Wellness” and “Patient Monitoring.” Importantly, user feedback provided exploratory, potentially valuable patient-reported outcome and experience information but is derived from personal opinions on “user experience,” “health improvement,” and “costs.” As quality information on DHTs could be identified in unstructured app metadata, this information should be improved in trustworthiness and more accessible to users like patients, supporting the public to find high-quality health apps. Furthermore, as user opinions contained information with potential value for patients, these should be explored as complementary value-generating indicators of health apps in future work.

## Author summary

People living with Alzheimer’s disease and Parkinson’s disease increasingly turn to mobile health apps to support their daily care and well-being. However, finding trustworthy, high-quality apps remains difficult, as app stores provide large amounts of information that are not always easy to interpret. In this study, we explored what information patients can access in app stores and whether it can help identify signals of value in health applications. We analyzed nearly 1,200 apps from Apple App Store and Google Play Store. We used artificial intelligence-assisted text analysis of app descriptions and user reviews to identify common themes, including patterns across different star ratings. We found that only about 2% of apps are certified medical devices, and many are not categorized as medical apps. Most patient-facing apps focus on care support, health and wellness, or patient monitoring. User reviews revealed exploratory insights into patient experiences, including usability, perceived health benefits, and costs. These findings suggest that app store information, particularly user feedback, contains potentially useful but currently underused signals about app quality. Making this information more accessible and easier to interpret could help patients and caregivers make better-informed decisions when choosing digital health tools.

## 1 Introduction

Alzheimer’s disease (AD) and Parkinson’s disease (PD) are the most common neurodegenerative conditions, with a rising prevalence and no curative treatment options. Given the complex pharmacological and non-pharmacological symptomatic treatment, there is an increasing demand for support services which can be potentially found in digital health technology (DHT) such as apps and others [1–5]. A rising number of mobile health and wellness applications aim to support clinical decision-making, diagnostic procedures, daily management, communication, and patient empowerment [6–13].

Ideally, people with AD/PD require apps that are user-centered and that are also responding to patients’ individual clinical and healthcare organizational needs [14–17]. Apple App store and Google Play store dominate the provision of apps [18], however, app stores provide limited information to users for identifying high-quality health and medical-related mobile apps [19]. Users such as patients can retrieve unstructured, limited and biased information on the quality of apps via app descriptions (metadata provided by manufacturers) and user feedback (provided by user ratings and reviews) [20,21].

A range of app information could suggest good quality of health apps – including digital medical device certification status (CE mark), description of app purpose, clinical characteristics, and target users, among others [22]. Furthermore, within the broad category of DHT, a new group of digital healthcare services, Digital Medical Devices (DMDs), may have undergone specific quality assessments [11,23–25]. Quality assessments include national health technology assessment (HTA) procedures, where quality is defined by “evidence-based medicine” (EBM). These quality assessment procedures aim to ensure safety and effectiveness of medical interventions and DHT/DMDs, and increasingly, their user-centeredness [14,25,26]. The HTA core model also addresses organizational aspects, patients and social aspects [27,28], and new HTA regulations and procedures increasingly emphasize the importance of user/patient engagement and empowerment in digital health technology related HTA procedures [29]. However, it is not defined in detail how user-centeredness and patient aspects could be “measured” and evaluated. One approach for EBM and HTA-related quality assessment is the evaluation of user-centeredness by Patient-Reported Outcome Measures (PROMs) and Patient-Reported Experience Measures (PREMs) [30–32]. PROMs capture the impact of interventions on patients’ health status, while PREMs assess the quality of care – both from patients’ perspectives. App stores provide user feedback as a prominent indicator of quality [33], leveraging perspectives of patients and other users on the user-centeredness of apps. This user feedback is known to have an impact on user-choices regarding apps [21,34,35], in absence of wide availability of more trusted information sources such as health professional recommendations [36], and despite that it is known to contain misinformation [37,38].

In this article, we aim to explore quality aspects of health app information provided by app stores and critically discuss their value and risks for app store users like AD/PD patients. To this end, we apply novel text mining approaches to explore and analyze app descriptions and user perspectives in reviews and ratings of AD/PD-related apps from Apple App store and Google Play store. To explore the availability of information on good app quality (c.f. [22]), we aim to identify information on medical device certification / CE mark, patient-facing category [39], application area and purpose, and, given the increasing relevance of user-centeredness [30–32], user opinions. We start with a descriptive statistical overview of app metadata based on a keyword search of apps that we retrieved using open-source APIs. Further, we present our content analysis, where we also explore novel text-mining approaches to app descriptions and user reviews, using Open AI GPT-4o large language models (LLMs) for content analysis, topic modelling for lexical topic exploration stratified by star ratings, and keyword-based approaches, informed by a literature search and manual labeling of the data. We critically discuss our findings in the light of evidence-based quality and user-centered personalized patient experience, forming a dual perspective on DHT value.

## 2 Materials and methods

We retrieved Parkinson’s and dementia app description/metadata (provided by manufacturers) and user feedback including ratings and reviews (provided by app users) from major app stores, Apple App Store (henceforth AA) and Google Play Store (henceforth GP). For this, we used the ‘google-play-scraper’ and ‘app-store-scraper’ open-source Python packages. We conducted step-by-step approaches to analyze the data, including a) a descriptive analysis of app metadata, b) exploratory content analysis of app descriptions and user reviews using OpenAI GPT-4o large language models (guided by literature search and manual data labeling), and c) a lexical topic exploration of user reviews stratified by star ratings. An overview of the steps taken is provided in Fig 1.

**Fig 1:**
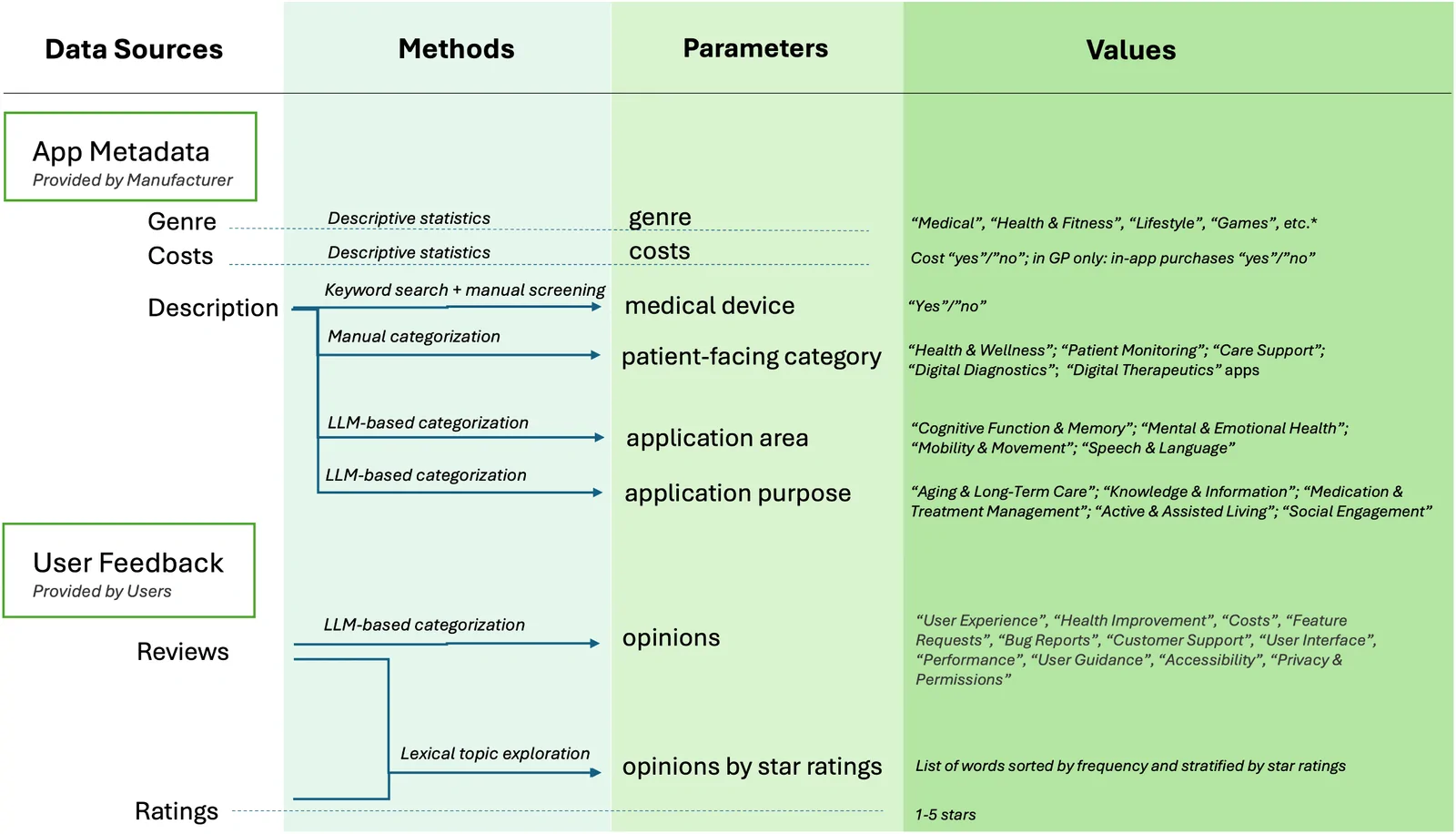
App metadata and user feedback was analyzed for quality information using descriptive statistics, manual categorization, LLM-supported approaches and topic modelling, which resulted in various output values.

### 2.1 Data retrieval: App metadata and user feedback scraping

We used open-source Python packages google-play-scraper and app-store-scraper to retrieve app metadata from GP (Google play store for Android apps) and AA (Apple app store for iOS apps), including ‘parkinson’, ‘alzheimer’, ‘dementia’, and ‘neurodegenerative’ keywords, and from 175 app store country codes (default for both scrapers was US). Using the scrapers, we retrieved metadata including app IDs, app name, country codes, seller name, artist id, artist name (AA)/developer name (GP), genre name (AA)/category (GP) – henceforth referred to as genre -; price and in-app purchases (GP) as cost-related data, and the manufacturer provided descriptions of the app. The source code for retrieving and analyzing the data set can be retrieved via the publicly accessible URL https://gitlab.com/uniluxembourg/lcsb/dmg/pdad-appstore. An app genre reflects an app category chosen by manufacturers, aiming to help users find apps according to their needs. For the user feedback analysis, we retrieved data from user ratings and user reviews. We also retrieved all user reviews for content analysis from the genres *Medical* and *Health & Fitness* released within 2018-01-06 and 2024-06-05 (the most recent reviews at the time of analysis) using the same scrapers. Apps assigned to these two genres by developers should aim to enhance medical care, treatment, medical education, and improve overall health outcomes; and help users to manage their health and fitness, promote healthy living, and health management [40,41].

### 2.2 Descriptive analysis of the app metadata

We used Python packages to comparatively analyze and plot the app metadata provided by manufacturers. Here, we conducted a descriptive analysis of the number of total and unique apps separated by keywords, genres, and app purchase costs.

Information on regulatory quality approvals such as certification as a medical device was not present as designated information in the metadata. According to recent research, however, information about CE mark is an important quality indicator for health apps [22]. Thus, we categorized apps as claiming to be a certified medical device if the following terms (translated into 30 different languages) could be identified through a keyword search in the description: ‘medical device’, ‘medical product’, ‘regulation’, ‘certified’, ‘certification’, and ‘FDA-cleared’. This was followed by a manual validation of the information.

For subgroup analysis, we selected apps in the *Medical* genre, which contains apps that aim to “enhance medical care, facilitate diagnosis and diagnosis and treatment, and improve overall health outcomes” [41]; focusing on “medical education, information management, or health reference for patients or healthcare professionals” [40]. We manually categorized the subgroup of apps into patient-facing categories (*Health & Wellness, Patient Monitoring, Care Support, Digital Diagnostics*, and *Digital Therapeutics* - guided by recent literature [39] -), based on textual information provided in the app descriptions.

### 2.3 Content analysis of app metadata using LLM-supported text mining

We further conducted a multi-step approach to classify apps in the *Medical* genre into different application areas and application purposes. This was informed by relevant literature, indicating that a description of app purpose and target user suggests good app quality [22]. We divided apps into application areas based on main clinical symptoms relevant in PD and AD disease ([42,43], S1 Table). The different application purposes we assigned to each app based on main patient-empowering self-management aspects in the disease ([44–50], S1 Table).

As the information provided from the meta-data category “descriptions” was not structured into these application categories and a simple screening for keywords was not feasible, we explored an LLM-based approach for text analysis that has gained increasing interest in digital health [51–54]. This computational approach allowed as to conduct an exploratory, hypothesis-generating classification of a larger set of metadata, where recent research suggests that the use of LLMs can support qualitative analysis of unstructured data [55]. A rationale for exploring the use of LLMs here is also to explore the extent to which app stores could automatize the classification of a larger number of apps based on the quality provided in unstructured metadata. For an exploratory content analysis of the app descriptions, we used of OpenAI GPT-4o API (https://openai.com/index/hello-gpt-4o/). We used the openai library in Python with which we connected to the API. In our Python code, we first read the csv file of app descriptions. We then used the following prompt to classify descriptions: “*Classify the following app description into one of these categories”*, followed by the nine categories and keywords in S1 Table and each app description, which we both joined with the prompt. We computed an exploratory classification of each app based on the metadata descriptions. We exported the results as a table and manually validated these by reading through the LLM-based classification. The outputs should be interpreted as exploratory classifications rather than validated automated assessments, given that no formal performance metrics were calculated.

### 2.4 Content analysis of user reviews using LLM-supported text mining

EBM procedures collect user experience and patient-centered outcome measures using structured assessment instruments to generate PROMs and PREMs. App-stores allow users to write user reviews in an unstructured way, leaving the content in terms of topic and evaluation free to the user. Thus, we applied a step-by-step approach to explore user “opinions” on AD/PD apps across user review content. Note, the analyzed reviews represent a subset of available user feedback at the time, rather than the overall population of app users or AD/PD patients. A total of N = 3181 (AA) and N = 681767 (GP) reviews from 46 AA apps and 45 GP apps were scraped and limited to English using the Python langdetect library (https://pypi.org/project/langdetect/), which resulted in a total of 690 reviews in AA, and 563151 reviews in GP. The 673 reviews in AA (*Medical* genre only) were manually assigned to an “opinion” category frequently used literature, such as user experience, health improvement, privacy and permissions, and others ([1,56–59], S2 Table). These 673 manually labeled reviews served to instruct the text-mining approach when applying the OpenAI GPT-4o LLMs. This approach allowed us to conduct an exploratory, hypothesis-generating classification of a larger set of user reviews, where recent research suggests that the use of LLMs can be support qualitative analysis of unstructured data [55]. A rationale for exploring the use of LLMs here is also to explore the extent to which app stores could automatize the classification of a larger number of unstructured user reviews. We used the openai library in Python with which we connected to the API. In our Python code, we first read the csv file of user reviews. We then used the following prompt to classify the reviews: “*Classify the following app review into one of these categories“*, followed by the opinion categories and their descriptions in S2 Table and each review, which we both joined with the prompt. An exploratory classification was performed on AA reviews (N=694) and a subset of GP reviews with a default limit (N=8400 based on computational feasibility and with reviews selected by app names in alphabetical order), generating an “opinion” category for each user review. Note, in order to classify a larger number of reviews, we also included reviews from the *Health & Fitness* genre in addition to those from the *Medical* genre, with user feedback on apps related to healthy living and health management [40,41]. We exported the results as a table and manually validated these by reading through the LLM-based classification. As also noted in section 2.3, the outputs should be interpreted as exploratory classifications rather than validated automated assessments, given that no formal performance metrics were calculated. Further, the subset of reviews analyzed from GP makes the approach an exploratory one, demonstrating the feasibility of such an LLM-supported classification.

### 2.5 Lexical topic exploration of user reviews stratified by star ratings

Another typical way to evaluate user feedback is to quantify the presence of words and terms used by users - ideally grouped into positive and negative feedback provided by users. This lexical topic exploration was conducted to quantify the most common words, a text-mining approach called topic modelling which has gained increasing interest in the field of digital medicine [43,60]. We first analyzed the user reviews descriptively by rating intervals according to their average stars – forming sets of negative (1–2), neutral (3), and positive (4–5) reviews. We then used the Python langdetect library to detect reviews in the English language. We converted the review text into lowercase to ensure consistency across the data. We removed special characters and numbers and removed stopwords specific to each language to filter out common, non-informative words. We utilized spacy’s language models (https://spacy.io/usage/large-language-models) to reduce words to their base or dictionary forms according to the specific linguistic rules. After preparing the user review data, we conducted our lexical topic exploration (using the topic modelling approach) to identify the most common words stratified by star ratings. To visualize the topic modelling applied to user reviews, we used the Python wordcloud library for computing word clouds. Doing so, we created a figure representing the frequency of words in user reviews on positively rated (4–5 stars) and negatively rated (1–2 stars) apps.

## 3 Findings

### 3.1 Total app search results

In May 2024, we retrieved in total of 1002 app search results in Apple App Store (AA), and 559 app search results in Google Play Store (GP) using the keywords ‘parkinson’, ‘dementia’, ‘neurodegenerative’, and ‘alzheimer’. These included 465 (AA) and 122 (GP) apps matching the keyword ‘dementia’, 368 (AA) and 98 (GP) apps matching ‘alzheimer’, 176 (AA) and 221 (GP) apps matching ‘parkinson’, and 1 (AA) and 118 (GP) apps matching ‘neurodegenerative’ (see Fig 2). Among these total results, which also included duplicates matching several keywords, we retrieved 798 (AA) and 439 (GP) unique apps in each app store. These included 50 apps available in both app stores, which resulted in a total of 1187 unique apps. As we are interested in the user/patient perspective and we assume app store users to access apps through either AA or GP and not both, we present our results separately for AA and GP.

**Fig 2:**
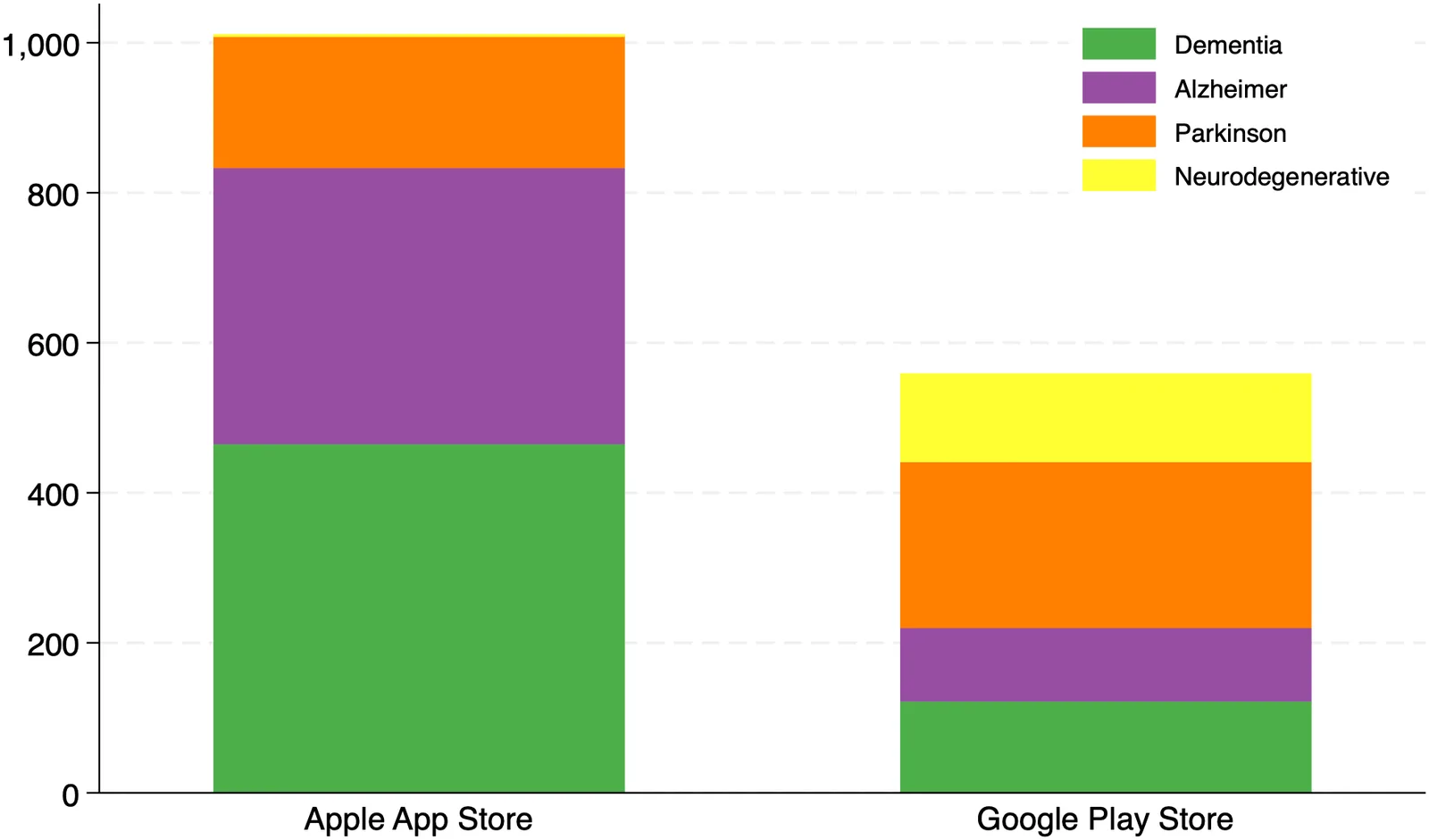
Number of app results by keywords in Apple App Store and Google Play Store, including duplicates. We retrieved 798 (AA) and 439 (GP) unique apps, including 50 apps available in both app stores, resulting in a total of 1187 unique apps.

### 3.2 App genres and payment status

The distribution of AD/PD-related apps across app genres was highly diverse spanning 21 genres in AA and 36 in GP (see Fig 3). Except for *Medical, Health & Fitness* and *Lifestyle* genres, the genres did not overlap across the two app stores (e.g., *Games* genre in GP includes 15 different Game_* sub-genres). Also, GP apps are subdivided into two levels of genres (primary and secondary genres). For the present analysis, we evaluated only the primary genre.

**Fig 3:**
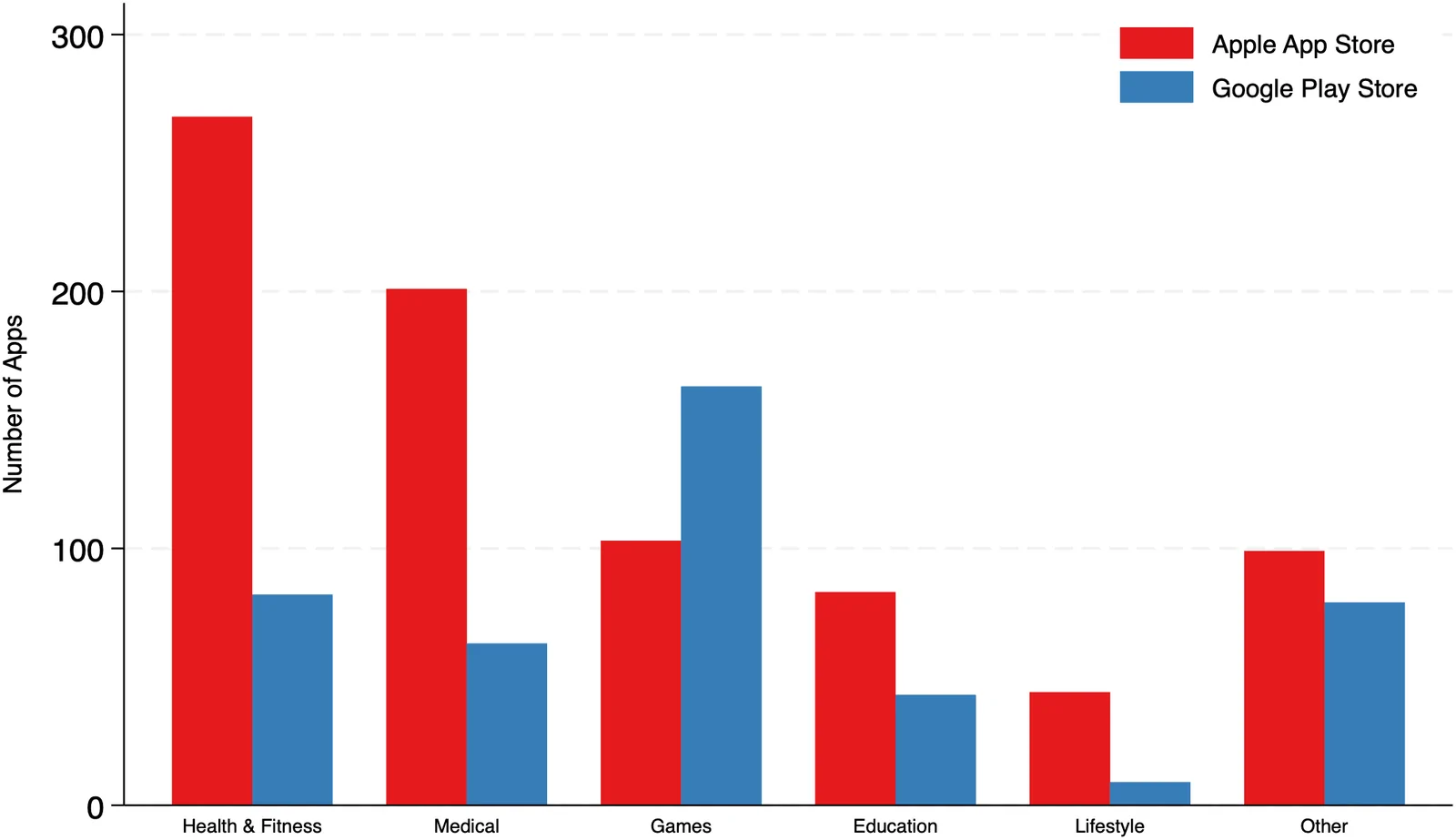
Number of apps by app store genre, summarized into six main genres.

In total, one third of AA apps vs. less than a fifth of GP apps belonged to the *Health & Fitness* genre, followed by roughly a fifth (AA) vs. a sixth (GP) in the *Medical* genre. While the majority (37.12%) of AA apps were categorized in a range of Game_* genres, merged in our figure in one single *Games* genre, approximately 13% of AA apps belonged to *Games* (AA). *Education* apps were roughly 11% (AA) and 10% (GP). The *Other* label in the figure includes 16 (AA) and 17 (GP) different genres, including less than 3% of apps. Notably, 63.08% of apps belonged to the three genres *Health & Fitness*, *Medical*, and *Lifestyle* in AA, but only 35,08% in GP.

Looking at the distribution of free and paid apps, out of 798 AA apps and 439 GP apps, 90.6% of AA apps and 97.7% GP apps claimed to be free (assuming other payment models, such as in-app purchases, advertisements, and others [61]). Additionally, 40.5% of GP apps (N = 178) claimed to have in-app purchases (i.e., a standard app version is free to download, but it contains additional functional components as premiums, that can be accessed for supplementary fees). No information on in-app purchases was given for AA apps by default.

### 3.3 Medical device grade information

As the medical device certification (CE) status was not an explicit term, genre, or category in the app metadata, we conducted a keyword search across the app descriptions of all results to identify apps which could potentially hold a CE mark. Among a total of 1187 apps (798 in AA, 439 in GP), we retrieved 57 apps which matched our keywords (40 in AA,17 in GP). After manually screening the 56 app descriptions, we found that 26 apps claimed to have medical device certification (18 in AA, 8 in GP) by description, matching in total only 2.2% of apps in AA and 1.8% in GP which communicated so to app store users.

### 3.4 Patient-facing categories of apps in the *Medical* genre

To understand the medical characteristics of the AD/PD-related apps, we analyzed the apps categorized in the *Medical* genre (containing apps that aim to “enhance medical care, facilitate diagnosis and diagnosis and treatment, and improve overall health outcomes” [41]; and focusing on “medical education, information management, or health reference for patients or healthcare professionals” [40]). Apps in this genre encompassed 201 (AA) and 63 (GP) apps in total. We quantified the apps in the medical genre as patient-facing apps/DHT by manually categorizing the provided app descriptions using categories defined in the literature [39]. For AA, we identified 63% patient-facing apps, with the majority (27%) dedicated to *Care Support*, followed by *Health and Wellness* and *Patient Monitoring* (both 16%). Among the 201 AA apps analyzed, only 1% of apps were categorized as *Digital Therapeutics*, and 3% as *Digital Diagnostics* (Fig 4). For GP, 54% of apps were patient-facing, with the majority (32%) being classified as *Care Support*, 13% as *Patient Monitoring*, and 8% as *Health and Wellness*. Among the 63 GP apps analyzed, only 1% of GP apps were a *Digital Diagnostics* app, and no app was classified as *Digital Therapeutics* app (Fig 4). Remarkably, several apps included diagnostic or therapeutic elements, however based on the literature [39], due to missing regulatory approval or scientific evidence, they were classified as *Care Support* or *Patient Monitoring*. Also, pure self-tracking apps without healthcare professional involvement were classified as *Care Support* apps, as by definition, *Patient Monitoring* apps entail communication pathways with healthcare professionals. This is due to the framework used which guided our manual categorization, according to which Digital Therapeutics and Digital Diagnostics apps require regulatory approval [39].

**Fig 4:**
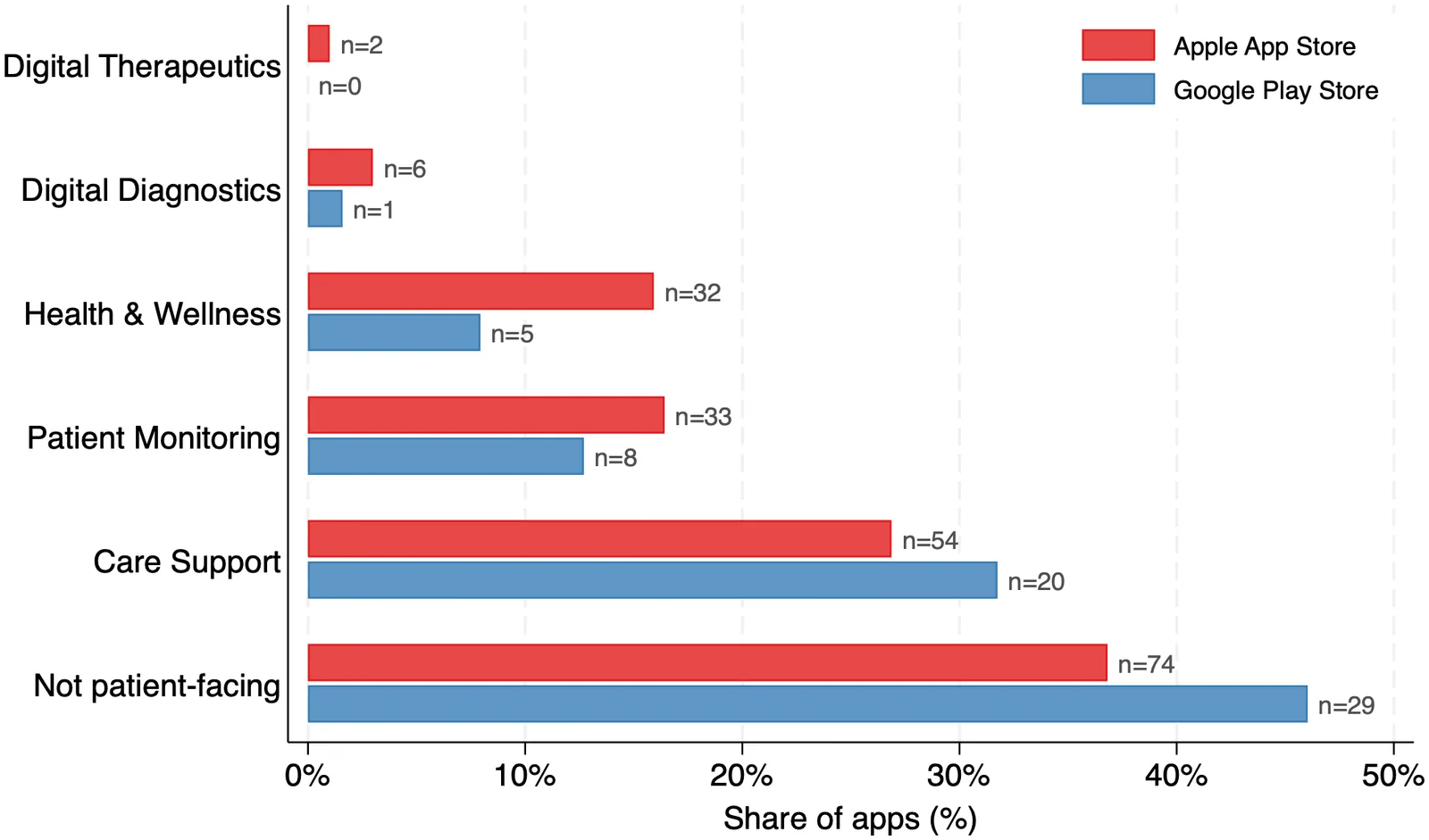
Descriptive distribution of apps in the “Medical” genre in the different patient-facing categories in Google play store (N = 34 apps) and Apple app store (N = 127 apps).

### 3.5 Clinical application area and organizational purpose of apps in the *Medical* genre

Since the above-mentioned categorization of patient-facing apps did not take into account any clinically relevant aspects related to the two specific diseases, we explored a classification of the main clinical and organizational needs of Alzheimer or Parkinson patients from the app description using OpenAI GPT-4o LLMs (publicly available, not open-source). We conducted an exploratory classification of the medical apps into eleven pre-defined clinical application areas and organizational purposes [42–50] – see Fig 5. In AA, the relative majority of apps in the *Medical* genre were classified as *Cognitive Function and Memory* (N = 44), followed by *Information and Education* (N = 40). In GP, the majority of apps in the *Medical* genre were on *Information and Education* (N = 30), followed by *Medication and Treatment Management* (N = 14).

**Fig 5:**
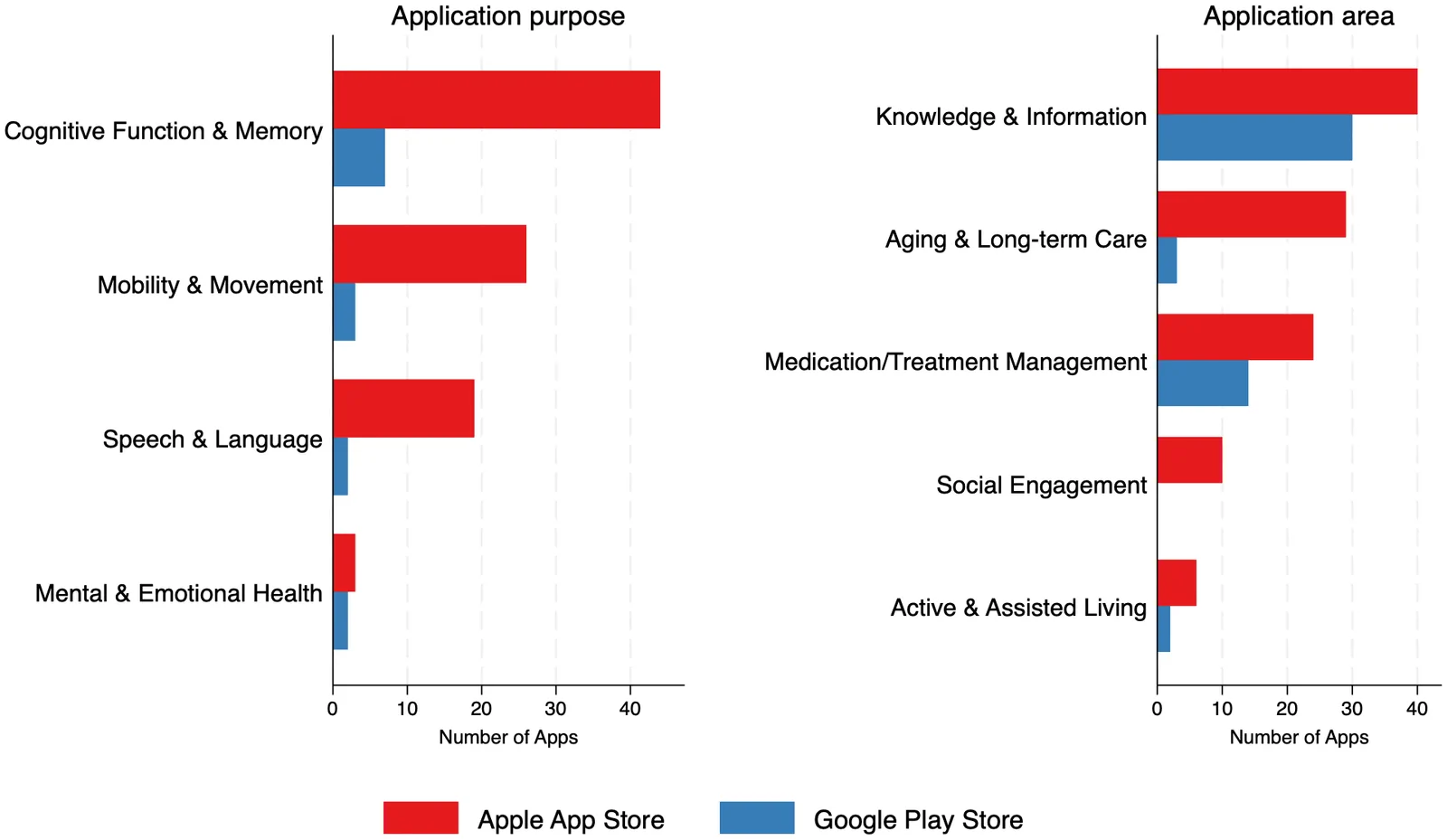
Classification of apps by application purpose (left plot) and application area (right plot). We analyzed apps in the Medical genre by description supported by OpenAI GPT-4o LLMs. In total, 61 Android and 201 iOS apps were included.

### 3.6 User feedback analysis

In addition to the metadata analysis representing the app information provided by manufacturers, we also explored user opinions by analyzing user ratings and review texts. To this end, we first manually assigned labels to AA reviews from the *Medical* genre. For this, we used predefined topics as defined through a literature search (S2 Table). Labels referred to topics such as accessibility, costs, health improvement, user experience, and others, as presented in detail in S2 Table.

We then conducted an exploratory AI-supported exploratory classification of English user reviews using LLMs (Fig 6), where we classified 258 *Medical* and 384 *Health & Fitness* apps of both AA (N = 634) and GP (N = 8400) app reviews. Here, the previously labeled AA reviews served to instruct the AI-supported classification. Note that we also included app reviews from the *Health & Fitness* genre to cover a broader perspective on opinions; given apps in this genre aim to promote healthy living and health management [40,41]. Our results show that the majority of users (here represented by a subset of available user feedback rather than the overall population of app users or patients) commented on *User Experience* in both AA and GP, followed by *Health Improvement/Medical Effect*. AA app review authors were also quite concerned about costs – despite 90.6% of apps being claimed to be free in total.

**Fig 6:**
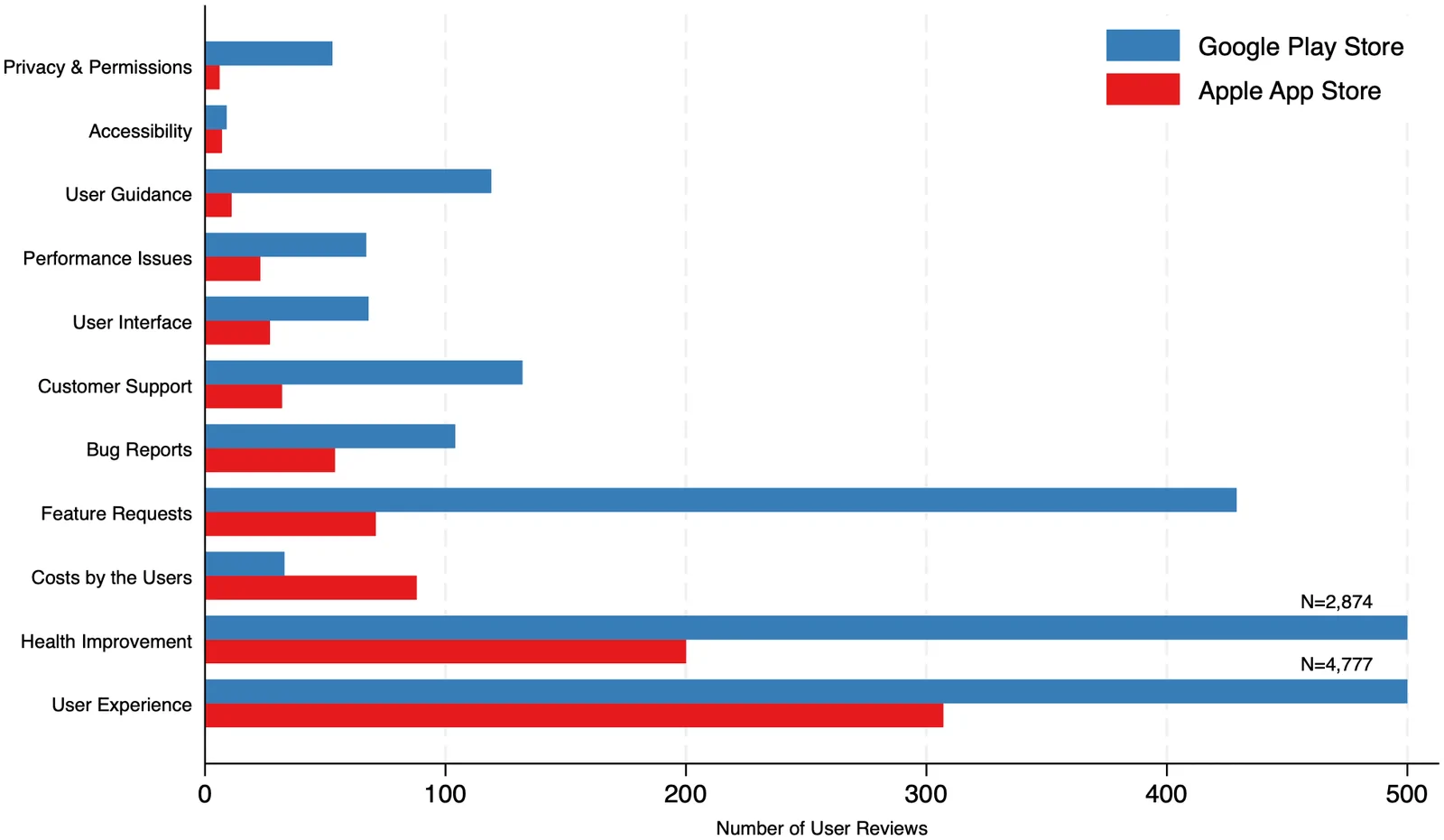
Classification of English user reviews using an AI-based approach to group user opinions by topic. With the support of LLMs, in total, 634 (AA) and 8400 (GP) user reviews were classified from the Medical (AA and GP) and Health & Fitness (AA) genre of apps.

In addition to the exploratory classification of user opinions, we conducted a lexical topic exploration of user reviews stratified by star ratings, using a topic modelling approach. We first analyzed user ratings descriptively by absolute number of reviews per star rating, as shown in Fig 7. On one hand, the GP apps contained significantly more ratings, i.e., 6152 ratings in GP vs. 3512 in AA, albeit with fewer apps in total (1006 AA vs. 441 GP apps). The absolute majority of user ratings was positive in both app stores, i.e., in the range of 4–5 stars, with 85.36% (N = 2998) in AA and 96.91% (N = 5962) in GP. In contrast, only 7.46% (N = 262) of ratings in AA, and 2.45% (N = 151) in GP were negative (i.e., 1–2 stars).

**Fig 7:**
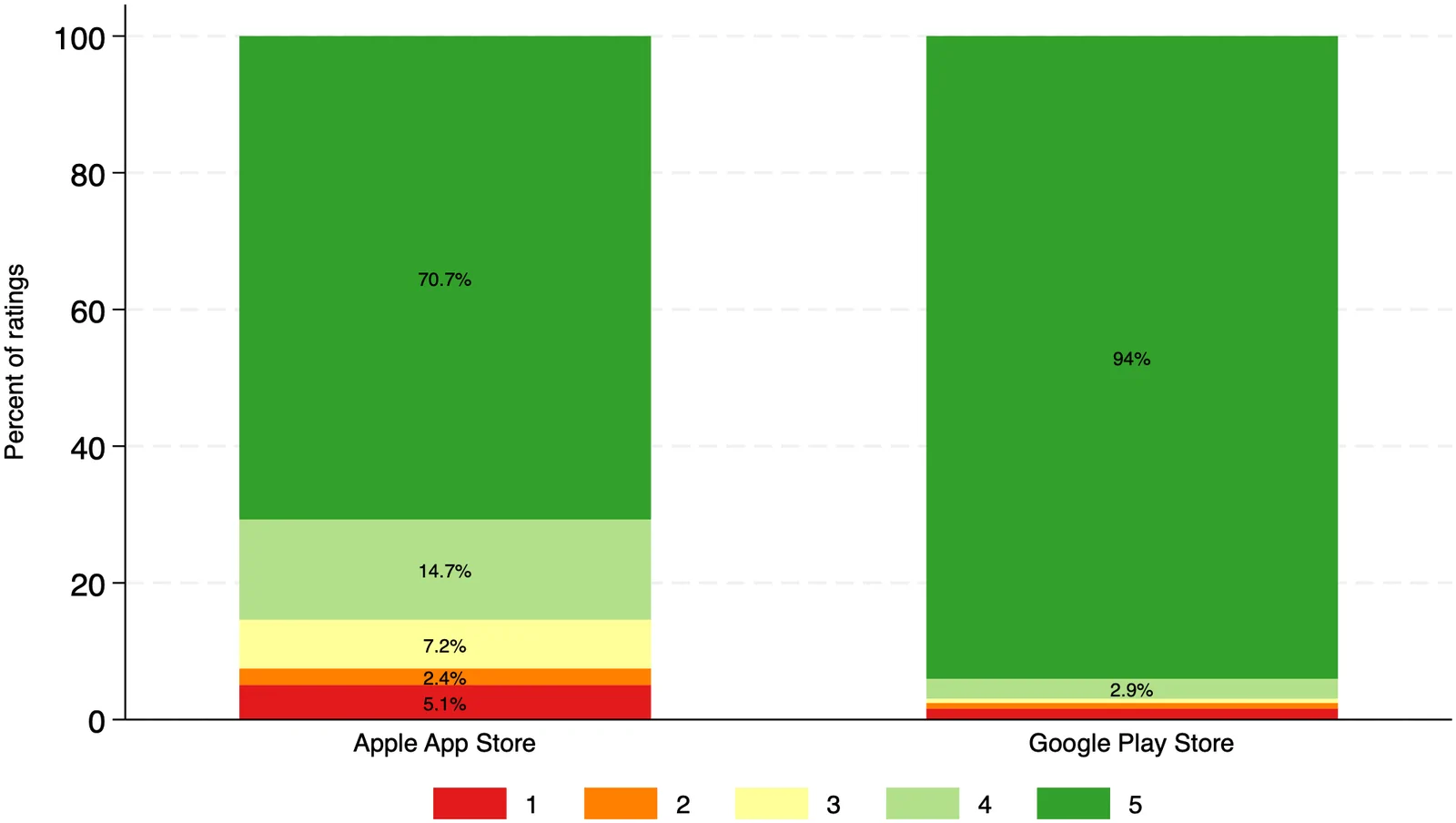
Positive trend of user ratings for all iOS (N = 1006) and Android (N = 441) search hits, also indicating a high popularity of the rating feature.

As a next step, word clouds were computed for positive, neutral, and negative user reviews in the English language. Content-wise, positive user reviews (Fig 8) contained superlatives and words referencing user experience (UX) aspects, such as *good, easy, great,* etc. in both app stores. We can also see, less prominently represented, health-specific words, such as *helpful, doctor, prescription,* etc. In negative user reviews, costs stand out in both app stores, represented by words like *free, pay, money*, etc.

**Fig 8:**
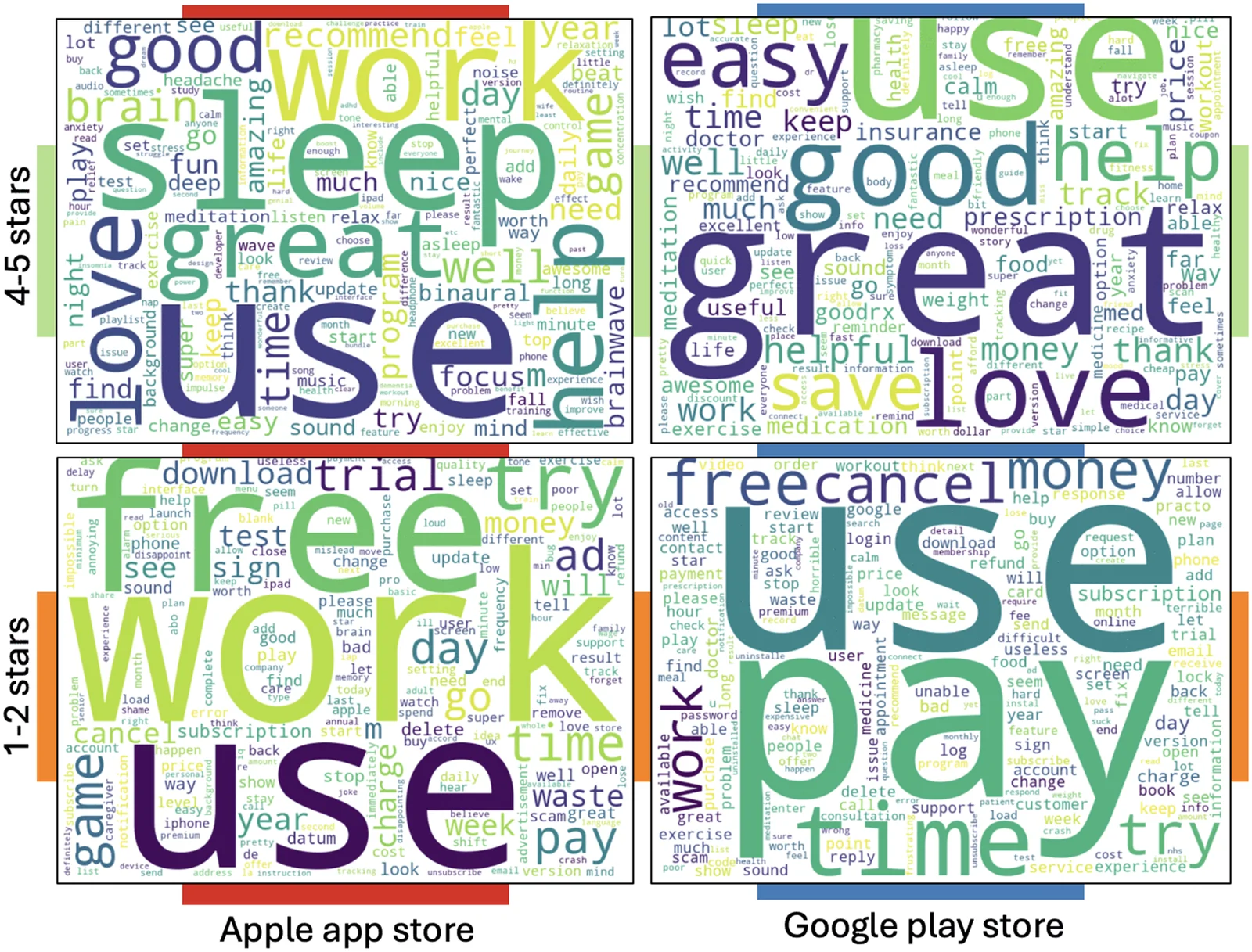
Word clouds of positive (upper part) and negative (lower part) user reviews in English from Apple app store (left) and Google play store apps.

## 4 Discussion

In the present article, we analyzed app store provided information for potential value-generating apps which might be suitable for AD/PD patients. We did this under the perspective of stratified quality information from an HTA/EBM perspective and user-centered experience reports on potential value provided by DHTs/DMDs. In total, we identified 1237 AD/PD-related apps which were found to be categorized into a total of 20 different app store genres (Fig 3). These included the *Medical, Health & Fitness* and *Lifestyle* genres in line with libraries recommended by EU bodies for medical device software (MDSW) [62] for these very large online platforms [63], but also various *Games* genres – especially in the Google Play Store (GP). Except for the three genres, *Medical*, *Health & Fitness* and *Lifestyle,* the genres differed between app stores both in numbers and in taxonomy. Roughly 24% (Apple) and 14% (Google) of apps were categorized into the *Medical* genre by their manufacturers.

As for information indicating good app quality (c.f. [22]) based on app descriptions/metadata, only ~ 2% of apps contained the information on being “certified medical devices” (holding a CE mark). By exploring an LLM-based approach, we identified key quality information content on application areas and purposes such as *Cognitive Function and Memory, Mobility and Movement,* and *Knowledge and Information* relevant for patients (Fig 5), but which were not provided in a structured or stratified manner in app stores. App descriptions in the *Medical* genre further contained information matching patient-facing categories (Fig 4) [39]. We categorized more than half of GP apps and almost two-thirds of AA apps in the *Medical* genre as “patient-facing”. . Remarkably, the absolute minority of patient-facing apps were *Digital Therapeutics* and *Digital Diagnostics* (1–2%), which may be also more challenging to develop given the regulatory procedures involved, compared to *Care Support* apps (~30%).

Overall, through an exploratory LLM-based classification and manual categorization of the provided metadata, we found that manufacturer-provided app descriptions contained quality information with potential relevance for patients, including clinical application areas and organizational application purposes, as well as patient-facing categories. The LLM-generated findings are of an exploratory nature, aiming to generate hypotheses rather than providing definite confirmatory statements about app store quality at the present time. Where the aim of this analysis is to describe currently available app information in app stores and discuss its potential value for patients in search for specific apps, the results also show that the current presentation of quality information is limited for users like patients who navigate the app stores to find a suitable app for their specific needs.

Through our analysis, we identified information from app descriptions indicating good app quality (c.f. [22]) such as information on primary user, clinical purpose, patient-facing category, and CE mark. Retrieving this quality information manually is however unfeasible for patients due to time-consuming procedures involved [57]. Therefore, patients currently face barriers in identifying high-quality health apps. A lack of user-friendliness in how high-quality health apps can be identified in app stores is a known challenge [19]. Structured presentation of quality information could enhance user-friendliness in identifying high-quality health apps – which would be especially relevant for patients to fit their individual needs and usage context (c.f. [15,17]). Structure could be added by implementing advanced filtering options (c.f. [19]). Apps could be filterable by primary user, application area and purpose, patient-facing categories, and evidence-based labels such as a CE mark or tangible benefits delivered that were confirmed by robust research (e.g., improved quality of life) which is also relevant to HTA procedures, such as application area or purpose. A structured presentation of this quality information would help to identify health apps that are safe and beneficial for self-care or healthcare delivery.

While we identified relevant quality information in app descriptions, a known challenge is that descriptions are biased by manufacturer interests and potentially unverified by app stores. Consequently, patients may be drawn to an app by its medically appealing description and use it without critical judgement, raising their expectations for tangible benefits [20,22]. Given that no significant quality control mechanisms in app stores currently exist for DHT/DMDs, there is a risk that an app may underdeliver due to a mismatch between over-selling descriptions and under-performance, diminishing user trust in DHT. Trustworthiness of quality information should therefore be improved. While our results show that only ~2% of apps among our search hits claimed to hold a CE mark, a comparison of our results with the EUDAMED database [64] could be undertaken in future work – verifying how many apps hold CE mark but do not claim so in the app store, or otherwise, how many claim to have CE mark but are not listed in the database. Moreover, users like patients may seek additional information on app quality via publicly available information such as app/manufacturer websites, which would be interesting to explore in future studies on quality information access. While CE mark signals high device quality, it remains largely complex, resource-intensive, and costly process, especially challenging for small and medium size enterprises [65]. As for app reimbursement, companies also face challenges in addressing HTA requirements [66,67], which require to demonstrate evidence-based quality such as safety and clinical effectiveness of DHT/DMDs (WHO, 2024; NICE ESF, 2023; [25,26]; Moshi et al., 2018). Nevertheless, if manufacturers manage to achieve both evidence requirements (for CE mark showing safety, and for HTA-related process showing clinical effectiveness), these could serve as strongest/most trustworthy indicators of app quality and should be explicitly highlighted in app descriptions. However, the high diversity of HTA frameworks currently in place, e.g., across European countries, limits scalability and broader market access [66,67]. To support broader adoption of quality assessments of DHT providing quality information of health apps, associated procedures should be more accessible and sustainable, and trustworthy quality information needs to be made available to app users like patients. This could be done by promoting quality labels designed for innovators and supporting their adoption [14]. Thus, HTA-related procedures and pathways to obtaining EBM quality standards and making this quality information available to patients should be further explored.

While the availability of structured quality information on health apps was limited, we identified a range of user opinions, which may help to assess app quality level and how patient-centered they are. User reviews and ratings are known to guide users in search for suitable apps [21,34] – even though prone to substantial biases coming from unstructured user-feedback and potentially false information. Using text mining approaches, we extracted and structured patient-reported feedback from user reviews and ratings. Using LLM-supported approach, we conducted an exploratory classification of user opinions by relevant topics such as user experience, health improvement, costs, and accessibility (Fig 6). Lexical topic exploration of user reviews stratified by star ratings further highlighted the different topics across differently rated apps: While reviews of negatively rated apps with 1–2 stars tended to emphasize costs and ads in reviews, reviews of positively rated apps with 4–5 stars tended to comment on overall user satisfaction (Fig 8). Given that EBM increasingly aims to evaluate user-centeredness using patient-reported measures (i.e. PROMs and PREMs) [30–32], we argue that user opinions point to interesting directions of future work, where their value for user-centeredness could be explored.

While our descriptive analysis of user ratings (Fig 7) indicates that the star-rating is a powerful and interactive tool, the overall high share of positive ratings suggests potential bias and provides little quality information on real DHT value, neither conveying any nuanced patient experiences. Current user ratings lack specificity and don’t reflect any needs of individual users, e.g., such as cognitive and physical impairments or accessibility needs potentially relevant to patients. Manual review screening is also time-consuming and biased [57,68]. As our analysis demonstrated, both user reviews and star ratings contain information that could potentially guide users in search for high-quality apps. Opinions could also be better structured in app stores, which provides an opportunity for a more personalized app search for patients. More detailed, feature-based, patient-centered rating systems (c.f. [68]) should be explored in the future, guided by user-centered topics, such as those classified in our user review analysis. Reviews could be filterable by topics relevant to a particular context, such as user experience, accessibility, perceived health improvement, and costs – enhancing user experience in identifying apps which are user-centered and tailored to individual needs. Future empirical research and participatory methods (co-design studies, structured user surveys (c.f. [24,69]) should also be applied to investigate the key filters/ factors that are the most important for users/patients when engaging with DHT and choosing suitable apps.

Concerning user reviews, text mining techniques (including AI-based ones) could be further explored to structure user reviews by topic, corresponding rating group, and user profiles to allow filtering of suitable reviews and support patients in identifying quality information provided by the peer-user community. Yet, such text-mining techniques would still depend on the quality of the review content, which may also be influenced by subjective and confounder factors related to review authors, and which may certainly be biased. To add transparency and improve quality, the overall trustworthiness of user feedback should be improved. This could be done, e.g., by identifying expert raters/reviewers, or by implementing credibility markers for trustworthy reviewers [34]. For patients to safely rely on quality information provided by other users, the critical aspect of rating/review trustworthiness clearly needs to be tackled.

In future work, the link between user opinions and EBM-guided quality assessment procedures should be explored. Certainly, user reviews represent available user feedback at the time of data collection and analysis, and these are not representative of the overall app user community or patient population. In our exploratory analysis, we focused on a subset of GP reviews published in English, which limits the generalizability of the findings to more linguistically diverse populations. Thus, our exploratory classification demonstrates the feasibility of this LLM-based approach rather than making any definite claims, especially given the reviews represent available user feedback at the time rather than a comprehensive patient population, and because only a subset of reviews was analyzed. Despite these limitations, HTA dimensions such clinical effectiveness, robustness, and user-centeredness were identified in our user review analysis, as reviews included comments on perceived health outcomes, costs, user satisfaction, and (perceived) technical quality. This points to very interesting areas of future work, where reviews could be analyzed more in-depth to identify quality information derived from user opinions concerning health improvement, user experience, costs, and others. Most importantly, they could be a complementary source of patient-generated health information and data. Patient-generated health information and data also becomes increasingly required for EBM quality assessments such as HTA procedures, i.e., as PROMs and PREMs (c.f. [30,32,70,71]). While LLM-supported content analysis as proposed here can be used to extract relevant user-centered content, it should be noted that this approach is fundamentally different from PROM/PREM methodologies, i.e., in terms of stratified selection of participants/patients, standardized assessment of information using validated self-reporting questionnaires, and study-design related monitoring procedures to ensure data quality in terms of completeness, errors (including duplication or fake information), etc. Thus, content analysis of user feedback has the potential to provide complementary information, but HTA or EBM-based evidence levels and procedures need to be further addressed. Certainly, user-generated reviews represent an exploratory source of patient perspectives that may complement but cannot replace structured and validated approaches used in HTA and EBM-based evaluation.

In summary, we provide a dual perspective on DHT value, based on analyzing information on potential app quality in app metadata and user reviews of Parkinson’s and dementia apps. As patients increasingly rely on app store-provided information on health apps, there is a big need to enhance patient access to quality. Through our mixed-methods analysis, we were able to identify and structure quality information from manufacturer-provided app descriptions and user feedback. While currently provided in a limited and unstructured format, such quality information could help less experienced app users to understand and infer app quality, and pick the right apps fitting their practical needs and clinical status. To help patients identify high-quality apps, trustworthy information should be provided in a structured format, including the filtering options for more effective information retrieval. App quality information should also be linked to EBM-guided quality assessments like HTA, where in future work, user opinions could be explored to provide complementary quality information on patient opinions and user-centeredness of health apps.

## Supporting information

S1 Table An overview of selected application categories (A: application area based on clinical symptoms; B: application purpose based on self-management aspects) that are relevant in Parkinson’s and Alzheimer’s disease. (DOCX)

S2 Table User opinions identified in 673 English iOS app reviews using manual labelling, along with a description and the number of reviews matching the topic. (DOCX)
