# Supplementary material for: Value in app store metadata and user reviews: A dual perspective on quality of Parkinson’s and dementia apps
Source: PLOS Digit Health. 2026 Oct 1;5(10):e0001730. doi: 10.1371/journal.pdig.0001730 (PMC13630307; doi:10.1371/journal.pdig.0001730)
Supplement: Supplementary file 1 [file pdig.0001730.s001.docx]

| **Application Category** |  |  |
| --- | --- | --- |
| **A: Application**  **area** | **Keywords matching relevant clinical symptoms** | **Ref.** |
| Cognitive Function & Memory | 'cognitive impairment', 'cognitive decline', 'dementia', 'memory loss', 'memory impairment', 'cognitive stimulation', 'cognitive training', ‘senior moment’, ‘memory’, ‘cognition’, ‘concentrate’, ‘dement’, ‘reaction time’, ‘concentration’, ‘reminiscent’, ‘cerebral aging’, ‘train your brain’, ‘cognitive function’, ’cognitive’, ‘brain power’, ‘brain training’, ‘brain’, ‘logical thinking’, ‘reaction’ | NINDS, 2025; Martin et al., 2013 |
| Mental & Emotional Health | 'mood swings', 'motivation', 'emotional stability', 'behavioral changes', 'hallucinations’, ‘mood’, ‘depression’, ‘anxiety’, ‘mental health’, ‘psychological’,  ‘anxious’, ‘mood swing’, ‘emotional stability’, ‘hallucinations’, ‘apathy’, ‘psychotherapy’, ‘sleep problem’, ‘sleep disorder’, ‘REM sleep’, ‘insomnia’, ‘sleep disorder’, ‘sleep quality’, 'sleep’, ‘sleep pattern’, ‘fatigue’ | NINDS, 2025; Tuijt et al., 2020;  Milne-Ives et al 2022 |
| Mobility & Movement | 'tremor', 'shaking', 'rigidity', 'muscle tension', 'bradykinesia', 'slowness', ‘fall detection’, ‘global mobility’, ‘GPS tracking’, 'instability', 'balance', 'gait', 'mobility’, ‘falling’, ‘trembling’, ‘dyskinesia’, ‘slow moving’, ‘physical impairment’, ‘dysphagia’, ‘swallowing disorder’ | NINDS, 2025; Tuijt et al., 2020; Milne-Ives et al. 2022 |
| Speech | 'speech difficulties', 'language impairment', 'communication challenges', 'voice issues’, ‘speech and language’, ‘speech therapy’, ‘language therapy’, ‘alphabet supplementation’, ‘difficulty speaking’, ’speech disorder’, ‘aphasia’, ‘speech production’, ‘voice recording’ | NINDS, 2025 |
| **B: Application purpose** | **Keywords matching relevant self-management aspects** | **Ref.** |
| Aging & Long-Term Care | 'aging effects', 'long-term care planning', 'caregiving challenges’, ‘care support’, ‘support caregiver’, ‘older adult care’, ‘family care’, ‘help caregiver’, ‘helps caregiver’, ‘support caregiver’, ‘caregiver stress’, ‘family member’, ‘app for caregiver’, ‘caregiving journey’ | Carros et al., 2022 ; Tuijt et al., 2020; Schwaninger et al., 2021 |
| Knowledge & Information | ‘prevention’, ‘health education’, ‘wellness’, ‘medical information’, ‘prevent’, ‘scientific information’, ‘publication’, ‘learn’, ’educational game’, provide knowledge’, ‘educational’, ’latest news’, ‘health information’, ‘medical simulation’, ’explain disease’, ‘information and resources’, ‘advice’, ‘self-learning’ | Bieg et al., 2022 ; Tuijt et al., 2020 |
| Medication & Treatment Management | 'medication reminders', 'treatment adherence', 'therapy sessions', 'management strategies', ‘treatment reminder’, ‘self-monitoring’, ‘tracking’, ‘diary’, ‘medical history questionnaire’, ‘changes in health status’ | Tuijt et al., 2020 |
| Active & Assisted Living | 'activities daily living', 'ADL', 'independence', 'home safety', ‘self-awareness’, ‘wellbeing’, ‘pain’, 'assistive technology’, ‘safe’, ’independent’, ‘geolocation’, ‘live independently’, ‘quality of home life’, ‘quality of life’, ‘nutrition’, ‘culinary’, ‘stress’, ‘medical alert’, ‘family members’, ‘emergency identification’, ‘emergency event’, ‘self-care’, ‘magnifier app’, ‘emergency situation’ | Bieg et al., 2022 ; Tuijt et al., 2020 |
| Social Engagement | ‘community building’, ‘community forum’, ‘participation’, ‘networking’, ‘stay in touch’, ‘support network’, ‘loved one’, ‘facilitate communication’, ‘facilitates communication’, ‘keep families connected’, ‘social isolation’, ‘assist them with caring’, ‘loneliness’, ‘stay in touch’, ‘families connected’ | Bieg et al., 2022; Schwaninger et al., 2020; Tuijt et al., 2020 |
