# Supplementary material for: Value in app store metadata and user reviews: A dual perspective on quality of Parkinson’s and dementia apps
Source: PLOS Digit Health. 2026 Oct 1;5(10):e0001730. doi: 10.1371/journal.pdig.0001730 (PMC13630307; doi:10.1371/journal.pdig.0001730)
Supplement: Supplementary file 2 [file pdig.0001730.s002.docx]

| **Opinion** | **Description** | **References** | **User Reviews** |
| --- | --- | --- | --- |
| Accessibility | Comments on app inclusiveness, and on how well an app accommodates users with disabilities or language barriers. | AlOmar et al., 2021; Haggag et al., 2022 ; Balaskas et al., 2022 | 27 |
| Bug Reports | Issues such as crashes, errors, and malfunctions that affect the app's functionality. | Malgaonkar et al., 2022 | 56 |
| Costs by the Users | Feedback regarding the pricing of the app, including affordability and value for money. | Balaskas et al., 2022; Haggag et al., 2022 | 71 |
| Customer Support | Reviews related to the quality and effectiveness of customer service and support provided by the app developers. | Balaskas et al., 2022 | 30 |
| Feature Requests | Suggestions for new features or improvements that users want to see added to the app. | Haggag et al., 2022; Malgaonkar et al., 2022 | 49 |
| Health Improvement/ Medical Effect | Insights into the app’s impact on health, including benefits, clinical effectiveness, and contributions to overall well-being. | Balaskas et al., 2022 | 110 |
| Performance Issues | Concerns about the app's speed, responsiveness, and overall performance. | Haggag et al., 2022; | 21 |
| Privacy and Permissions | Concerns about data privacy, security, and how permissions are managed by the app. | Alhammad et al., 2024; Haggag et al., 2022; Van den Berg et al., 2025 | 3 |
| User Experience | Overall user satisfaction, as well as ease of use, interaction with the app, intuitiveness, and fit to everyday life. | Haggag et al., 2022 | 499 |
| User Guidance | Comments on the clarity and usefulness of instructions, manuals, and support materials within the app. | Balaskas et al., 2022 | 24 |
| User Interface | Feedback on the app’s visual design, layout, graphical presentation, its usability and aesthetics. | Balaskas et al., 2022; Haggag et al., 2022 | 46 |
